# Comparative effectiveness of non-invasive interventions for stage I post-stroke shoulder–hand syndrome: a network meta-analysis of randomized controlled trials

**DOI:** 10.3389/fneur.2026.1932931

**Published:** 2026-09-23

**Authors:** Xiao-Zhong Liu, Ruo-Yang Li, Ke-Yu Chen, De-Liang Zhu

**Affiliations:** 1 Department of Rehabilitation, Geriatric Diseases Institute of Chengdu, Chengdu Fifth People's Hospital, The Second Clinical Medical College, Affiliated Fifth People's Hospital of Chengdu University of Traditional Chinese Medicine, Chengdu, China; 2 Department of Traditional Chinese Medicine, Chengdu Second People's Hospital, Chengdu, Sichuan, China

**Keywords:** network meta-analysis, non-invasive, randomized controlled trials, shoulder hand syndrome, stroke

## Abstract

**Background:**

To compare the effectiveness of non-invasive interventions for pain, upper-limb motor function, and activities of daily living in patients with stage I post-stroke shoulder–hand syndrome (SHS) using network meta-analysis.

**Methods:**

Randomized controlled trials evaluating non-invasive interventions for stage I SHS were systematically retrieved. Outcomes included visual analogue scale (VAS), Fugl–Meyer Assessment–Upper Extremity (FMA-UE), and Barthel Index (BI). Mean differences (MDs) or standardized mean differences (SMDs) with 95% confidence intervals (CIs) were estimated, and surface under the cumulative ranking curve (SUCRA) values were calculated.

**Results:**

Thirty-six RCTs involving 2,769 participants were included. The VAS, FMA-UE, and BI networks included 24, 28, and 18 RCTs, respectively. Compared with conventional treatment (CT), VAS scores were significantly reduced by kinesiology taping (KT) (MD = −1.95, 95% CI − 2.85 to −1.05), air pressure therapy (AT) (MD = −2.30, −4.31 to −0.29), Traditional Chinese Medicine External Herbal Therapy (TCM-EHT) (MD = −1.36, −2.08 to −0.64), Traditional Chinese Medicine External Physical Stimulation (TCM-EPS) (MD = −1.43, −2.36 to −0.50), and moxibustion (MD = −1.52, −2.53 to −0.51). FMA-UE improved with TCM-EPS (SMD = 2.12, 0.03 to 4.21), TCM-EHT (SMD = 2.07, 0.62 to 3.52), and KT (SMD = 2.32, 0.04 to 4.61). BI improved with TCM-EHT (SMD = 3.64, 1.76 to 5.51), TCM-EPS (SMD = 2.55, 0.60 to 4.50), and KT (SMD = 1.98, 0.27 to 3.70). However, 95% prediction intervals for all statistically significant comparisons crossed the null. AT, KT, and TCM-EHT ranked highest for VAS, FMA-UE, and BI, respectively. No statistically significant global inconsistency was detected, although localized disagreement was identified for CT versus TCM-EHT in the VAS network.

**Conclusion:**

Non-invasive interventions showed outcome-specific potential benefits, but no intervention demonstrated consistent superiority across all outcomes. Although AT and KT ranked favorably for pain and KT, TCM-EPS, and TCM-EHT for functional outcomes, wide prediction intervals indicated substantial uncertainty. Given methodological limitations and imprecision, findings should be interpreted cautiously and confirmed by adequately powered multicentre RCTs.

**Systematic review registration:**

The publicly accessible registration record is available through the PROSPERO database: https://www.crd.york.ac.uk/PROSPERO/view/CRD420251276361. The systematic review was registered in PROSPERO (registration number: CRD420251276361).

## Introduction

Stroke is a leading cause of death and long-term disability among adults worldwide. With continuous improvements in acute management and secondary prevention, the survival rate of patients with stroke has increased substantially; however, post-stroke functional impairments have become increasingly prominent. Post-stroke shoulder–hand syndrome (SHS) is a common complication after stroke and shares several clinical characteristics with complex regional pain syndrome type I (CRPS I), including pain, oedema, autonomic dysfunction, and motor impairment. Although SHS has historically been regarded as a post-stroke manifestation related to CRPS-like syndrome, it represents a stroke-associated clinical entity and should not be considered completely equivalent to CRPS I. It most frequently occurs during the early stage after stroke (1, 2). From a pathophysiological perspective, SHS is generally regarded as a result of multiple interacting factors following central nervous system injury. Previous studies have suggested that stroke-related impairment of central nervous system regulation may lead to autonomic dysfunction, particularly sympathetic imbalance, resulting in peripheral vasomotor dysregulation, microcirculatory disturbances, and tissue oedema, which constitute an important pathological basis for SHS development. Additionally, neurogenic inflammation plays a critical role in its onset and progression, as abnormal release of inflammatory mediators and neuropeptides may enhance peripheral nociceptive sensitivity and contribute to a persistent pain state (3, 4). Stage I SHS is mainly characterized by shoulder and hand pain, oedema, alterations in skin temperature and color, restricted joint mobility, and upper limb motor dysfunction, which severely impair upper limb functional recovery and activities of daily living (5). Evidence suggests that if stage I SHS is not treated in a timely and standardized manner, the condition may progress further, leading to chronic pain, joint contracture, and irreversible functional impairment, thereby significantly delaying overall rehabilitation and reducing quality of life (6).

Currently, management of SHS relies mainly on noninvasive interventions. Commonly used clinical approaches include conventional therapy (CT), physical agent therapies, such as ultrashort-wave therapy (UW) and extracorporeal shock wave therapy (SW), air pressure therapy (AT), mirror therapy (MT), motor imagery therapy (MIT), negative-pressure mechanical stimulation therapy (NPMST), kinesiology taping (KT), and manual lymphatic drainage (MLD). In addition, various TCM-related external therapies, such as herbal applications, cupping, and moxibustion, have been increasingly applied in clinical rehabilitation settings (7–9). However, considerable heterogeneity exists among available studies regarding patient characteristics, diagnostic criteria, intervention components, treatment dosage, treatment duration, and the content of conventional rehabilitation provided in control groups. These variations have resulted in inconsistencies across study findings, leading to a lack of consensus regarding the relative effectiveness of different noninvasive interventions in clinical practice.

Previous systematic reviews and meta-analyses have provided valuable evidence regarding non-pharmacological interventions for post-stroke SHS; however, most previous studies relied on conventional pairwise meta-analysis, which generally evaluates the comparative effects between only two interventions. In the clinical context of stage I SHS, where multiple non-invasive rehabilitation interventions are often applied in parallel, traditional meta-analyses are limited in their ability to simultaneously compare the relative effects of multiple treatment strategies. Therefore, clinicians may face challenges in selecting appropriate interventions when direct head-to-head comparisons between different treatment options are unavailable. Moreover, when different studies employ different control interventions, the conclusions drawn from pairwise meta-analyses may have limited value in guiding clinical decision-making (10). Network meta-analysis (NMA), as an extension of conventional meta-analysis, enables the integration of both direct and indirect evidence within a unified statistical framework, allowing simultaneous comparisons and ranking of multiple interventions. Compared with traditional pairwise meta-analysis, NMA provides additional information regarding the relative effectiveness and ranking probabilities of multiple interventions, which may facilitate a more comprehensive understanding of available treatment options. Although several NMAs have been conducted in China to evaluate various non-invasive therapies for post-stroke SHS, most previous analyses included patients across different disease stages. Considering that clinical manifestations, rehabilitation priorities, and potential treatment responses may vary during different stages of SHS, a stage-specific evaluation focusing on stage I SHS remains clinically relevant. Furthermore, interpretation of NMA findings requires consideration of potential effect modifiers. These factors may include time since stroke onset, stroke severity, baseline pain and motor impairment, oedema severity, diagnostic criteria, intervention dosage, treatment duration, and the content of conventional rehabilitation.

Based on this background, the present study systematically searched for and included previously published randomized controlled trials (RCTs) investigating non-invasive interventions for Stage I SHS. An NMA was conducted to evaluate the comparative effectiveness of different noninvasive interventions for stage I SHS. Pain intensity assessed by the visual analogue scale (VAS), upper limb motor function evaluated using the Fugl–Meyer Assessment of the upper extremity (FMA-UE), and activities of daily living measured by the Barthel Index (BI) were selected as primary outcomes. By comprehensively assessing the relative effects and rankings of different interventions compared with CT, as well as among the interventions themselves, this study aimed to evaluate the comparative effectiveness and relative ranking of different non-invasive interventions for stage I SHS, while providing insights into the uncertainty and limitations of the current evidence base.

## Method

### Registration

This systematic review and network meta-analysis was registered in the International Prospective Register of Systematic Reviews (PROSPERO; registration number: CRD420251276361) on 31 December 2025. The registration included the review objectives, eligibility criteria, planned outcomes, and data synthesis methods. Any deviations from the registered information during the review process are described in Supplementary Table 1.

### Search strategy

A comprehensive search strategy combining controlled vocabulary terms and free-text terms was developed for each database. All databases were searched from inception to July 2026, and the search was updated before manuscript submission. The following electronic databases were systematically searched: PubMed, Embase, the Cochrane Central Register of Controlled Trials, China National Knowledge Infrastructure (CNKI), Wanfang Data, Chinese Biomedical Literature Database (CBM), and Weipu Database. Additional sources were searched to identify potentially eligible studies, reference lists of included studies and previous systematic reviews, and citation tracking of eligible studies. Grey literature sources identified through Chinese databases, including dissertations and conference records, were screened according to the predefined eligibility criteria. Complete search strategies for all databases, including controlled vocabulary terms, free-text terms, and Boolean operators, are provided in Supplementary File 1.

### Eligibility criteria

Studies were included if they met the following criteria: Study design: RCTs investigating non-invasive interventions for stage I SHS were eligible, regardless of whether allocation concealment or blinding was reported; Participants: Adults with stroke confirmed by neuroimaging examinations (e.g., computed tomography or magnetic resonance imaging) and clinically diagnosed with post-stroke SHS were eligible. The diagnostic criteria used for SHS and stage I classification in each included study were extracted and recorded; Disease stage: Studies involving patients with stage I SHS at baseline were included. When different staging criteria were reported, the definitions and classification methods were recorded for comparison; Interventions and comparators: Eligible interventions included non-invasive treatments for stage I SHS, administered either alone or in combination with conventional therapy (CT). Eligible comparator interventions included CT alone or other single non-invasive interventions. The components, intensity, frequency, and duration of CT and other comparator interventions were extracted and reported where available; Outcomes: Studies reporting validated measures of pain intensity, upper limb motor function, or activities of daily living were eligible. Specific measurement instruments were not used as mandatory inclusion criteria. When different instruments assessed the same clinical outcome construct, appropriate effect measures were applied for data synthesis.

### Exclusion criteria

Studies were excluded if they were reviews, cohort studies, animal experiments, case studies, basic research, cross-sectional studies, or case reports; if they did not report relevant clinical outcomes related to pain intensity, upper limb motor function, or activities of daily living; if the full text was unavailable, relevant data could not be extracted or complete original data were not provided, and attempts to obtain missing data from the authors were unsuccessful; if the studies were duplicate publications; or if the interventions involved multiple non-invasive interventions that could not be separated into individual treatment effects or were not comparable within the predefined network nodes.

### Study selection and data extraction

Two trained reviewers independently screened the literature and extracted the data using a standardized data extraction form. An initial screening was conducted based on titles and abstracts, followed by a full-text review of the studies that met the inclusion criteria. When necessary, the corresponding authors were contacted to obtain missing or unclear information and minimize data omission. Any discrepancies between the reviewers were resolved through discussion with a third reviewer. EndNote and Microsoft Excel were used for literature management and data extraction, respectively. The extracted data included study characteristics (first author, publication year, country, and study design), participant characteristics (sample size, age, sex, stroke duration, stroke severity when available, and SHS diagnostic criteria), disease stage definitions, intervention characteristics (treatment components, dosage, frequency, duration, and details of conventional therapy), and outcome information. For each extracted outcome, the original reported values, calculation procedures, and any assumptions used for data transformation were recorded. For all continuous outcomes, change scores (i.e., post-treatment values minus baseline values) were used for analysis. When change scores were not reported in the original studies, they were calculated using a standard formula (11), assuming a correlation coefficient of 0.5 between baseline and post-treatment measurements. The assumptions and procedures used for data transformation were recorded during data extraction.


$$SD_{E\;\text{change}}=\sqrt{\begin{array}{l} SD_{E\;\text{baseline}}^{2}+SD_{E\;f\text{inal}}^{2}-\\ \left(2\times \text{Corr}\times SD_{E\;\text{baseline}}\times SD_{E\;\text{final}}\right) \end{array}}$$

$$\mathrm{Mea}n_{E\;\text{change}}=\mathrm{Mea}n_{E\;\text{final}}-\mathrm{Mea}n_{\begin{array}{c} E\;\text{baseline} \end{array}}$$

### Treatment classification and network node definition

Treatment nodes were defined according to the predominant therapeutic mechanism and clinical mode of delivery. Moxibustion was treated as an independent node because of its distinct thermal mechanism. Mechanically based external stimulation techniques, including cupping, scraping, meridian-based plucking, and related manipulative stimulation, were grouped as Traditional Chinese Medicine External Physical Stimulation (TCM-EPS), whereas external herbal interventions, including herbal fumigation, soaking, hot compresses, and related topical herbal therapies, were grouped as Traditional Chinese Medicine External Herbal Therapy (TCM-EHT). Other interventions with distinct therapeutic mechanisms were retained as separate nodes.

### Risk of bias assessment

The risk of bias of the included randomized controlled trials was assessed independently by two reviewers using the Cochrane Risk of Bias 2 (RoB 2) tool recommended in the updated Cochrane Handbook for Systematic Reviews of Interventions. The RoB 2 tool evaluates five domains: bias arising from the randomization process, bias due to deviations from intended interventions, bias due to missing outcome data, bias in measurement of the outcome, and bias in selection of the reported result. Each domain was judged as “low risk of bias”, “some concerns”, or “high risk of bias”. The overall risk of bias judgment was determined for each included study based on the domain-level assessments, and supporting reasons were recorded. Any disagreements were resolved through discussion with a third reviewer.

### Statistical analysis

Network meta-analysis was performed using STATA software (version 14.0; Stata Corporation, Lakeway, TX, USA) within a multivariate random-effects consistency framework. A common between-study heterogeneity variance was assumed across treatment comparisons and estimated using restricted maximum likelihood (REML), with correlations arising from multi-arm trials accounted for within the multivariate framework. For continuous outcomes, mean differences (MDs) were used for VAS because the included studies used comparable pain scales. Standardized mean differences (SMDs) were used for FMA-UE because differences in the reporting and scoring ranges of the Fugl–Meyer assessment were identified across studies, despite all eligible studies assessing upper-extremity motor function using the Fugl–Meyer framework. SMDs were also used for the BI network because different versions of the Barthel Index, including the modified Barthel Index (MBI), were reported. Positive effect estimates for FMA-UE and BI indicated greater improvement in motor function and activities of daily living, respectively, whereas negative MDs for VAS indicated greater pain reduction. The transitivity assumption was assessed qualitatively by examining available information on clinically relevant study and participant characteristics across treatment comparisons, including stroke type, time since stroke, diagnostic and staging criteria, and the content of conventional rehabilitation. Because several potentially relevant effect modifiers were incompletely or inconsistently reported across studies, formal meta-regression or quantitative assessment of effect-modifier distributions was not feasible. STATA was used to generate network plots, league tables, forest plots, ranking probability plots, and comparison-adjusted funnel plots. Treatment uncertainty was assessed using 95% prediction intervals (PrIs), which were interpreted together with effect estimates and 95% confidence intervals (CIs). Global inconsistency was evaluated by comparing consistency and inconsistency models, whereas local inconsistency was assessed using loop-specific inconsistency factors (IFs) and node-splitting analysis. When potential sources of inconsistency were identified, sensitivity analyses were performed by excluding relevant studies and repeating the network meta-analysis. The surface under the cumulative ranking curve (SUCRA) was used to estimate the relative ranking probabilities of interventions. SUCRA results were interpreted together with treatment effects, confidence intervals, prediction intervals, and certainty of evidence rather than as direct evidence of clinical superiority. The certainty of evidence for the main network comparisons was assessed using the Confidence in Network Meta-Analysis (CINeMA) framework, considering within-study bias, reporting bias, indirectness, imprecision, heterogeneity, and incoherence. The certainty of evidence was classified as very low, low, moderate, or high.

## Results

### Study selection

A total of 7,516 records were identified through the literature search. After removing duplicates, 4,536 records were excluded, leaving 2,980 records for screening. After title and abstract screening, 2,838 records were excluded. After a full-text review, additional 106 studies were excluded. Ultimately, 36 studies were included in the final analysis (12–47). A total of 15 treatment strategies were used: CT, TCM-EPS, MIT, TCM-EHT, KT, MLD, AT, MT, UW, NPMST, suspension exercise therapy (SET), SW, warm low-frequency therapy (WLF), Moxibustion and LT. The literature search process and study selection results are presented in Figure 1. The full-text assessment results and reasons for exclusion are provided in Supplementary Table 2.

**Figure 1:**
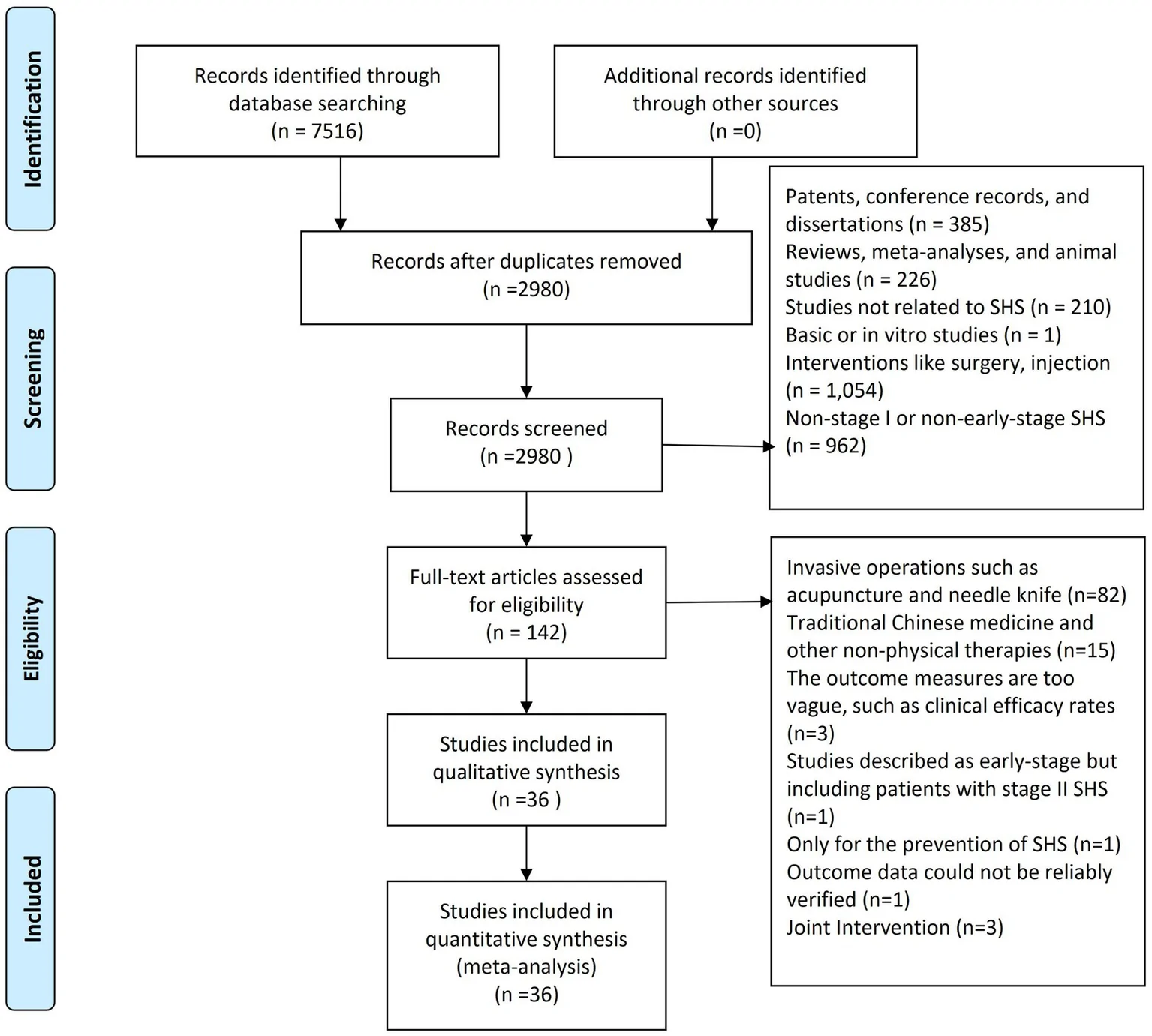
Literature search process.

### Study characteristics

The 36 included studies (12–47) were published between 2012 and 2025 and included a total of 2,769 participants. In all the included studies, the intervention groups underwent CT combined with a single noninvasive intervention. Among the included trials, 24 reported pain outcomes assessed using the VAS, 28 reported upper limb motor function assessed using the FMA, 18 reported activities of daily living assessed using the BI. The basic characteristics of the included studies are summarized in Table 1 and Supplementary Table 3. The detailed data extraction table is provided in Supplementary Table 7.

**Table 1:** Basic characteristics of trials included.

Author	Year	Number of patients			Age(year)			Male/female	Treatment			Intervention period	Outcome indicator
		I		C	I		C		I		C		
Cai YH (12)	2022	30		30	59.26 ± 7.58		59.7 ± 5.98	40/20	CT + TCM-EPS (Cupping)		CT	2 W	VAS, FMA, BI
Chen RQ (13)	2020	61		60	64.28 ± 6.23		61.38 ± 7.47	68/53	CT + TCM-EHT		CT	2 W	FMA
Chen YH (14)	2024	30	30	30	66.16 ± 1.72	62.27 ± 2.33	67.32 ± 2.44	62/28	CT	CT + KT	CT + TCM-EHT	4 W	VAS, FMA, BI
Fu Y (15)	2025	30		30	57.97 ± 6.23		55.53 ± 11	33/27	CT + Moxibustion		CT	2 W	VAS, BI
He QC (16)	2013	60		61	65.25 ± 9.97		64.7 ± 8.37	76/45	CT + Moxibustion		CT	4 W	FMA, BI
He LY (17)	2023	30		30	56.5 ± 6.86		56.6 ± 7.9	25/35	CT + TCM-EPS (Gua sha)		CT	2 M	VAS, BI
Hu KH (18)	2012	30		30	59 ± 4.8		58 ± 4.2	33/27	CT + AT		CT	4 W	VAS
Huang WL (19)	2023	30		30	51.69 ± 5.12		52.66 ± 4.25	36/24	CT + Moxibustion		CT	2 M	FMA
Li H (20)	2019	40		40	62.2 ± 8.9		61.7 ± 9.3	47/33	CT + UW		CT	4 W	FMA, BI
Li H (21)	2020	30	30	30	56.72 ± 5.33	55.67 ± 6.21	57.43 ± 4.54	/	CT	CT + Moxibustion	CT + MLD	4 W	VAS, BI
Li HZ (22)	2018	30		30	55–68		52–65	28/32	CT + KT		CT	4 W	VAS, FMA, BI
Li JJ (23)	2021	30		30	49.79 ± 3.46		55.34 ± 1.78	27/33	CT + TCM-EPS (Cupping)		CT	24D	VAS, FMA
Li K (24)	2022	30		30	59.65 ± 6.78		58.83 ± 7.05	33/27	CT + MLD		CT + KT	4 W	VAS, FMA, BI
Li L (25)	2017	23		23	56.43 ± 7.91		57.09 ± 8.07	28/18	CT + TCM-EPS (Suspended meridian-following plucking therapy)		CT	8 W	VAS, FMA
Li XJ (26)	2023	30		30	65.7 ± 16		64.97 ± 7.06	35/25	CT + TCM-EHT (Traditional Chinese medicine iontophoresis)		CT	2 W	VAS, FMA
Ou LZ (27)	2016	30		30	58.73 ± 6.74		58.95 ± 7.16	34/26	CT + WLF		CT	4 W	FMA
Qiao XQ (28)	2020	32		28	60.5 ± 3.83		59.34 ± 5.03	45/15	CT + TCM-EHT		CT	2 W	VAS, FMA, BI
She LY (29)	2013	30		30	57.39 ± 3.18		59.13 ± 4.53	34/26	CT + UW		CT	4 W	VAS
Shen Z (30)	2024	40		40	69.23 ± 5.39		66.53 ± 6.58	52/28	CT + MIT		CT + TCM-EHT	2 W	VAS, FMA, BI
Song YJ (31)	2024	20		20	44.35 ± 10.287		51.05 ± 10.081	34/6	CT + MT		CT	3 W	VAS, FMA
Sun F (32)	2018	31		31	54 ± 12.48		53.18 ± 11.88	35/27	CT + UW		CT	3 W	VAS, FMA
Tan LL (33)	2015	27		27	54.1 ± 6.3		55.2 ± 5.6	28/26	CT + TCM-EHT (Acupoint ironing)		CT	21D	FMA, BI
Wang LY (34)	2021	39		39	69.12 ± 4.98		68.49 ± 5.1	45/33	CT + MLD		CT + SW	4 W	VAS, FMA
Wu JX (35)	2025	31		31	55.46 ± 6.26		57.84 ± 6.61	40/22	CT + TCM-EHT		CT	4 W	FMA
Wu M (36)	2018	40		40	56.85 ± 6.6		57.85 ± 6.49	48/32	CT + KT		CT	4 W	FMA, BI
Wu SH (37)	2017	30		30	54.5 ± 6.5		56.2 ± 7.6	34/26	CT + AT		CT	18D	FMA, BI
Xu ZQ (38)	2015	52		52	49.21 ± 13.21		51.3 ± 12.9	54/50	CT + Moxibustion		CT	22D	VAS, FMA
Yang ZW (39)	2017	163		163	58.3 ± 11.9			176/150	CT + KT		CT	3 W	VAS
You GJ (40)	2022	30		30	59.58 ± 5.86		57.46 ± 6.23	27/33	CT + NPMST		CT	4 W	VAS, FMA, BI
Yu C (41)	2013	25		25	60 ± 1.3		61 ± 1.2	37/13	CT + TCM-EPS (Medicated stick acupoint therapy)		CT	4 W	VAS, FMA, BI
Yu LL (42)	2015	30		30	60.45 ± 7.57		60.1 ± 6.85	35/25	CT + TCM-EHT		CT	2 W	FMA
Zhang JP (43)	2019	29		29	53.91 ± 5.53		53.7 ± 5.73	28/30	CT + SET		CT + SW	2 W	VAS, FMA
Zhang LY (44)	2022	43		43	61.71 ± 7.22		61.84 ± 7.13	32/54	CT + TCM-EHT		CT	4 W	VAS
Zhang ZP (45)	2023	40		40	74.4 ± 12.6		70.3 ± 13.5	47/33	CT + TCM-EHT		CT	3 W	FMA
Zhao XK (46)	2021	51		50	58.12 ± 2.41		57.89 ± 2.37	54/47	CT + LT		CT	28D	FMA, BI
Zhu S (47)	2025	30		30	60.21 ± 3.49		59.83 ± 3.2	33/27	CT + TCM-EHT		CT	3 W	VAS

### The quality assessment of the included studies

Risk of bias assessments were performed using the Cochrane Risk of Bias 2 (RoB 2) tool for each outcome-specific study result. Overall, most included studies were judged as having some concerns regarding risk of bias, mainly due to insufficient reporting of allocation concealment, limited information regarding blinding procedures, and potential concerns related to selective reporting. For the VAS outcome, among the 24 included RCTs, 22 were judged as having some concerns and 2 were judged as having a high risk of bias. For FMA-UE, 26 of the 28 included RCTs were judged as having some concerns and 2 were judged as having a high risk of bias. For BI, all 18 included RCTs were judged as having some concerns, with no studies classified as high risk of bias. Across outcomes, concerns were most frequently observed in the domains related to randomization procedures, deviations from intended interventions, measurement of outcomes, and selection of the reported results. For subjective outcomes such as VAS, concerns regarding outcome measurement were particularly common because blinding of participants and personnel was generally difficult to achieve in non-invasive rehabilitation interventions. The detailed risk of bias assessments are presented in Supplementary Table 4.

### VAS

#### Network geometry

A total of 24 studies reported VAS outcomes involving 13 treatment strategies. In the network plot, the size of the nodes represents the sample size for each intervention, and the thickness of the edges indicates the number of RCTs that directly compare the two corresponding interventions. Three closed loops were identified in the evidence network. The network geometry of the VAS is shown as Figure 2A.

**Figure 2:**
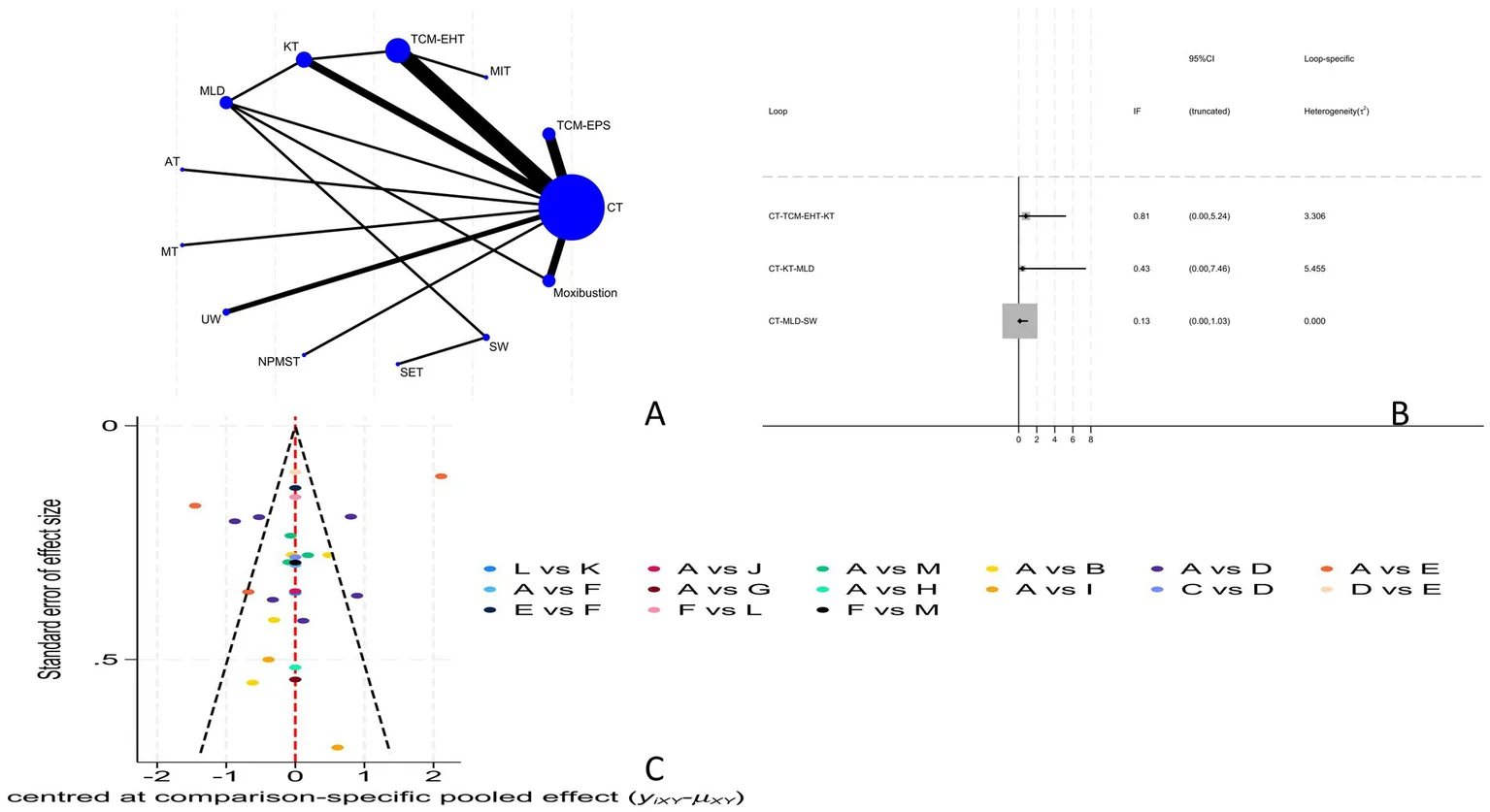
**(A)** Network diagram of VAS. **(B)** VAS inconsistency testing. **(C)** Publication bias graph of VAS.

#### Inconsistency assessment

For VAS, the 13 treatment strategies formed three triangular loops. The global inconsistency test showed no statistically significant evidence of inconsistency (*p* = 0.299). Loop-specific analysis likewise showed no statistically significant inconsistency in the CT-TCM-EHT-KT loop (IF = 0.81, 95% CI 0.00–5.24), the CT-KT-MLD loop (IF = 0.43, 95% CI 0.00–7.46), or the CT-MLD-Moxibustion loop (IF = 0.14, 95% CI 0.00–1.03). However, node-splitting analysis identified statistically significant disagreement between direct and indirect evidence for the CT versus TCM-EHT comparison (direct MD = −1.23, indirect MD = −4.72, *p* = 0.042), whereas no statistically significant disagreement was detected for the remaining evaluable comparisons. The details are shown in Figure 2B and Table 2.

**Table 2:** Node splitting result of VAS.

Side	Direct		Indirect		Difference		*p*
	Coef.	Std. Err.	Coef.	Std. Err.	Coef.	Std. Err.	
A D*	−1.225651	0.3371938	−4.724245	1.687686	3.498594	1.723285	0.042
A E	−2.023082	0.5367115	−1.665263	1.029056	−0.3578186	1.163464	0.758
A F	−0.360013	0.9485189	−0.7750592	0.9360295	0.4150462	1.332527	0.755
A M*	−1.493055	0.5431357	−2.272807	2.660236	0.7797515	2.715474	0.774
C D*	−0.2	0.9144772	−2.524456	199.9365	2.324456	199.939	0.991
D E	0.1684634	0.8617655	−1.065454	0.6872804	1.233918	1.102789	0.263
E F	1.199998	0.9113007	1.590244	1.006556	−0.3902461	1.357801	0.774
F L*	−0.1100004	0.8834809	0.3759172	89.41658	−0.4859175	89.42089	0.996
F M	−1.029511	0.9499954	−0.8340431	1.1995	−0.1954682	1.529773	0.898
K L*	−0.36	0.9408098	−1.003111	200.0063	0.6431112	200.0092	0.997

A, CT; B, TCM-EPS; C, MIT; D, TCM-EHT; E, KT; F, MLD; G, AT; H, MT; I, UW; J, NPMST; K, SET; L, SW; M, Moxibustion (* all the evidence about these contrasts comes from the trials which directly compare them).

To explore the robustness of the localized disagreement observed for the CT versus TCM-EHT comparison, leave-one-study-out sensitivity analyses were performed. Exclusion of Li et al. (26), Zhang et al. (44), or Qiao et al. (28) attenuated the node-splitting signal to borderline non-significance (*p* = 0.055, 0.051, and 0.051, respectively), whereas exclusion of Zhu et al. (47) did not materially reduce the inconsistency (*p* = 0.031). Exclusion of Chen et al. (14) substantially altered the network geometry and resulted in an extremely imprecise indirect estimate for the CT versus TCM-EHT comparison (SE ≈ 100), limiting the interpretability of the corresponding node-splitting result. Overall, no single study clearly accounted for the observed localized incoherence.

#### Network meta-analysis

The network consisted of 13 intervention nodes: CT, TCM-EPS, MIT, TCM-EHT, KT, MLD, AT, MT, UW, NPMST, SET, SW, and moxibustion. Compared with CT, TCM-EPS (MD = −1.43, 95% CI − 2.36 to −0.50), TCM-EHT (MD = −1.36, 95% CI − 2.08 to −0.64), KT (MD = −1.95, 95% CI − 2.85 to −1.05), AT (MD = −2.30, 95% CI − 4.31 to −0.29), and moxibustion (MD = −1.52, 95% CI − 2.53 to −0.51) were associated with statistically significant reductions in VAS scores. In contrast, MIT, MLD, MT, UW, NPMST, SET, and SW did not show statistically significant differences compared with CT. Most pairwise comparisons among active interventions were also not statistically significant. The network estimates with corresponding 95% confidence intervals and 95% prediction intervals are presented in Table 3 and Figure 3. Notably, the 95% prediction intervals for all interventions compared with CT crossed the null value, indicating considerable uncertainty regarding the treatment effects that might be expected in future comparable settings.

**Table 3:** Network meta-analysis of VAS.

Treatment	Moxibustion	SW	SET	NPMST	UW	MT	AT	MLD	KT	TCM-EHT	MIT	TCM-EPS	CT
Moxibustion	-												
SW	−0.84 (−3.08,1.39)	-											
SET	−1.20 (−4.10,1.69)	−0.36 (−2.20,1.48)	-										
NPMST	−0.25 (−2.35,1.85)	0.59 (−2.24,3.41)	0.95 (−2.43,4.32)	-									
UW	−0.14 (−1.92,1.63)	0.70 (−1.90,3.29)	1.06 (−2.12,4.24)	0.11 (−2.24,2.46)	-								
MT	−0.47 (−2.70,1.75)	0.37 (−2.55,3.29)	0.73 (−2.73,4.18)	−0.22 (−2.93,2.49)	−0.33 (−2.79,2.13)	-							
AT	0.78 (−1.47,3.02)	1.62 (−1.32,4.56)	1.98 (−1.49,5.45)	1.03 (−1.70,3.76)	0.92 (−1.56,3.40)	1.25 (−1.57,4.07)	-						
MLD	−0.95 (−2.36,0.46)	−0.11 (−1.84,1.62)	0.25 (−2.28,2.78)	−0.70 (−2.93,1.54)	−0.81 (−2.74,1.12)	−0.48 (−2.83,1.87)	−1.73 (−4.10,0.65)	-					
KT	0.42 (−0.87,1.72)	1.27 (−0.89,3.42)	1.63 (−1.21,4.46)	0.68 (−1.37,2.73)	0.57 (−1.15,2.28)	0.90 (−1.28,3.08)	−0.35 (−2.56,1.85)	1.38 (0.10,2.66)^%^	-				
TCM-EHT	−0.16 (−1.40,1.07)	0.68 (−1.56,2.92)	1.04 (−1.86,3.94)	0.09 (−1.89,2.07)	−0.02 (−1.65,1.61)	0.31 (−1.80,2.42)	−0.94 (−3.07,1.20)	0.79 (−0.63,2.22)	−0.59 (−1.65,0.48)	-			
MIT	−0.36 (−2.54,1.81)	0.48 (−2.39,3.35)	0.84 (−2.57,4.25)	−0.11 (−2.78,2.56)	−0.22 (−2.64,2.20)	0.11 (−2.66,2.88)	−1.14 (−3.93,1.65)	0.59 (−1.70,2.88)	−0.79 (−2.87,1.30)	−0.20 (−1.99,1.59)	-		
TCM-EPS	−0.10 (−1.47,1.28)	0.75 (−1.59,3.09)	1.11 (−1.87,4.09)	0.16 (−1.91,2.22)	0.05 (−1.68,1.78)	0.38 (−1.81,2.57)	−0.87 (−3.09,1.34)	0.86 (−0.71,2.43)	−0.52 (−1.82,0.78)	0.07 (−1.11,1.25)	0.27 (−1.88,2.41)	-	
CT	−1.52 (−2.53,-0.51)^%^	−0.68 (−2.83,1.46)	−0.32 (−3.15,2.51)	−1.27 (−3.11,0.57)	−1.38 (−2.84,0.08)	−1.05 (−3.03,0.93)	−2.30 (−4.31,-0.29)^%^	−0.57 (−1.84,0.69)	−1.95 (−2.85,-1.05)^%^	−1.36 (−2.08,-0.64)^%^	−1.16 (−3.09,0.77)	−1.43 (−2.36,-0.50)^%^	-

%There is a statistical difference between the two in comparison.

**Figure 3:**
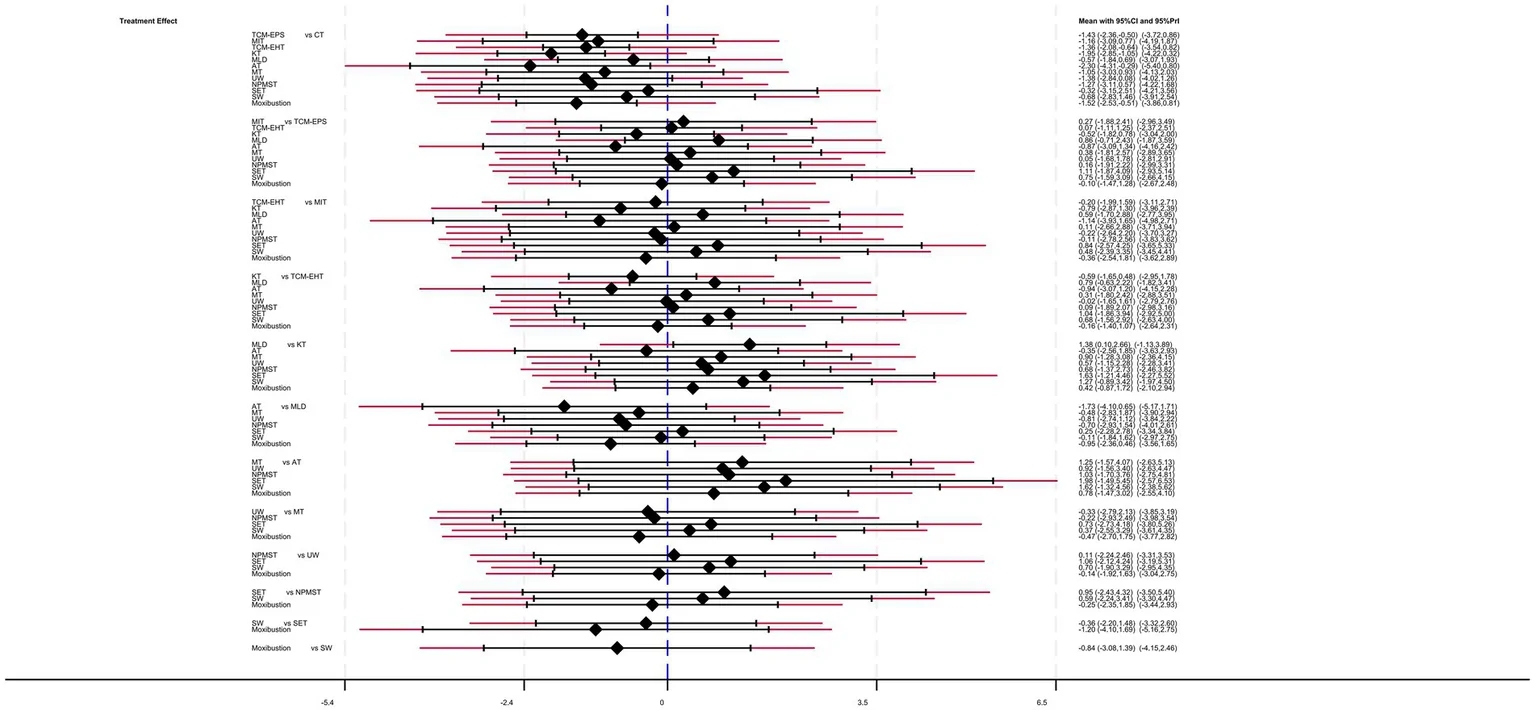
Forest plot of VAS.

#### Publication bias

The comparison-adjusted funnel plot for the VAS network did not show pronounced overall asymmetry, although several estimates were relatively dispersed. Given the limited number of studies contributing to some treatment comparisons and the sparsity of the network, small-study effects or publication bias could not be excluded. The comparison-adjusted funnel plot for VAS is shown in Figure 2C.

### FMA-UE

#### Network geometry

28 studies reported FMA-UE outcomes involving 15 treatment strategies. Two closed loops were identified in the evidence network. The network geometry of the FMA-UE is shown in Figure 4A.

**Figure 4:**
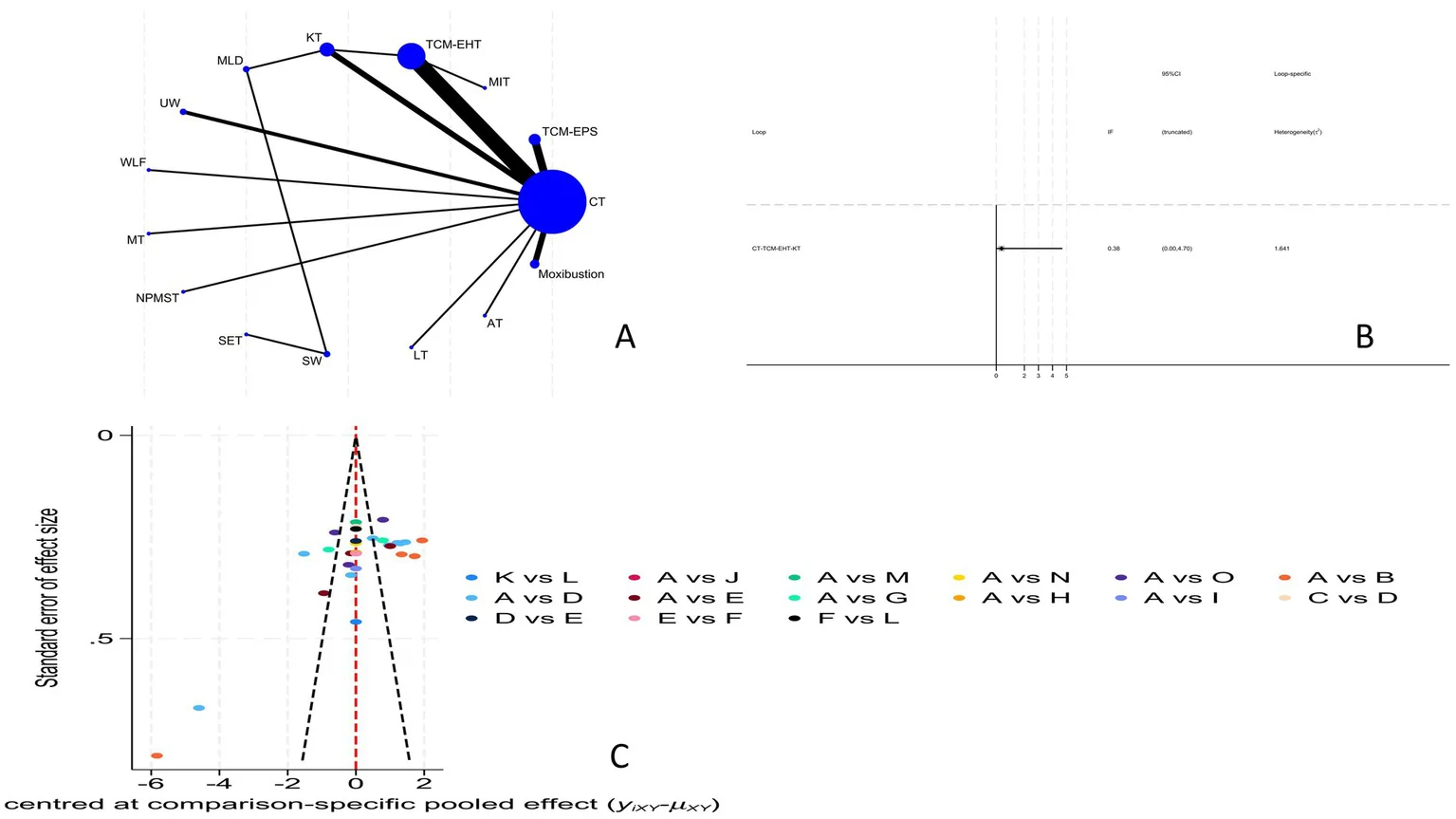
**(A)** Network diagram of FMA-UE. **(B)** FMA-UE inconsistency testing. **(C)** Publication bias graph of FMA-UE.

#### Inconsistency assessment

No statistically significant evidence of global inconsistency was detected in the FMA-UE network (*p* = 0.7775). Loop-specific analysis likewise showed no statistically significant inconsistency in the available closed loop; the CT–TCM-EHT–KT loop had an inconsistency factor of 0.38 (95% CI 0.00 to 4.70) (Figure 4B). Node-splitting analysis did not identify statistically significant disagreement between direct and indirect estimates for any evaluable comparison (all *p* > 0.05). Overall, no statistically significant evidence of either global or local inconsistency was observed in the FMA-UE network.

#### Network meta-analysis results

A total of 28 RCTs contributed to the FMA-UE network meta-analysis, comprising 15 treatment nodes: CT, TCM-EPS, MIT, TCM-EHT, KT, MLD, UW, WLF, MT, NPMST, SET, SW, LT, AT, and moxibustion. Higher FMA-UE scores indicated better upper limb motor function. Because differences in FMA scoring and reporting ranges were identified across studies, treatment effects were expressed as standardized mean differences (SMDs). Compared with CT, statistically significant improvements in upper limb motor function were observed with TCM-EPS (SMD = 2.12, 95% CI 0.03 to 4.21), TCM-EHT (SMD = 2.07, 95% CI 0.62 to 3.52), and KT (SMD = 2.32, 95% CI 0.04 to 4.61). In contrast, MIT, MLD, UW, WLF, MT, NPMST, SET, SW, LT, AT, and moxibustion did not show statistically significant differences compared with CT. Pairwise comparisons among all interventions are presented in the league table. The network estimates with corresponding 95% confidence intervals and 95% prediction intervals are shown in Table 4 and Figure 5. Although TCM-EPS, TCM-EHT, and KT showed statistically significant improvements compared with CT, their corresponding 95% prediction intervals crossed the null value (TCM-EPS: −2.98 to 7.22; TCM-EHT: −2.75 to 6.89; KT: −2.88 to 7.52), indicating substantial uncertainty regarding the expected effects in future comparable studies.

**Table 4:** Network meta-analysis of FMA-UE.

Treatment	Moxibustion	AT	LT	SW	SET	NPMST	MT	WLF	UW	MLD	KT	TCM-EHT	MIT	TCM-EPS	CT
Moxibustion	-														
AT	1.12 (−3.63,5.88)	-													
LT	0.69 (−4.06,5.43)	−0.44 (−6.25,5.38)	-												
SW	1.36 (−5.32,8.05)	0.24 (−7.24,7.72)	0.68 (−6.80,8.15)	-											
SET	5.25 (−2.64,13.13)	4.12 (−4.45,12.69)	4.56 (−4.01,13.12)	3.88 (−0.30,8.07)	-										
NPMST	0.37 (−4.39,5.13)	−0.75 (−6.58,5.07)	−0.32 (−6.14,5.50)	−0.99 (−8.48,6.49)	−4.88 (−13.45,3.70)	-									
MT	1.04 (−3.73,5.81)	−0.09 (−5.92,5.75)	0.35 (−5.48,6.18)	−0.32 (−7.82,7.17)	−4.21 (−12.79,4.37)	0.67 (−5.17,6.51)	-								
WLF	0.41 (−4.35,5.17)	−0.71 (−6.54,5.11)	−0.28 (−6.10,5.54)	−0.95 (−8.44,6.53)	−4.84 (−13.41,3.74)	0.04 (−5.79,5.87)	−0.63 (−6.47,5.21)	-							
UW	0.47 (−3.29,4.23)	−0.66 (−5.70,4.39)	−0.22 (−5.25,4.81)	−0.90 (−7.79,6.00)	−4.78 (−12.84,3.28)	0.10 (−4.95,5.15)	−0.57 (−5.63,4.48)	0.06 (−4.99,5.11)	-						
MLD	0.86 (−4.42,6.13)	−0.27 (−6.52,5.99)	0.17 (−6.08,6.42)	−0.51 (−4.61,3.60)	−4.39 (−10.25,1.47)	0.49 (−5.77,6.75)	−0.18 (−6.45,6.09)	0.45 (−5.81,6.71)	0.39 (−5.15,5.93)	-					
KT	−0.55 (−3.84,2.74)	−1.68 (−6.38,3.03)	−1.24 (−5.94,3.46)	−1.92 (−7.73,3.90)	−5.80 (−12.96,1.36)	−0.92 (−5.63,3.79)	−1.59 (−6.31,3.13)	−0.96 (−5.67,3.75)	−1.02 (−4.72,2.68)	−1.41 (−5.53,2.71)	-				
TCM-EHT	−0.30 (−3.08,2.49)	−1.42 (−5.78,2.94)	−0.98 (−5.34,3.37)	−1.66 (−8.01,4.69)	−5.54 (−13.14,2.06)	−0.67 (−5.04,3.70)	−1.34 (−5.72,3.04)	−0.71 (−5.08,3.66)	−0.76 (−4.01,2.49)	−1.15 (−5.99,3.69)	0.26 (−2.28,2.80)	-			
MIT	−0.02 (−4.98,4.95)	−1.14 (−7.13,4.85)	−0.70 (−6.69,5.28)	−1.38 (−8.94,6.18)	−5.26 (−13.90,3.38)	−0.39 (−6.38,5.61)	−1.06 (−7.06,4.95)	−0.43 (−6.42,5.57)	−0.48 (−5.72,4.76)	−0.87 (−7.22,5.48)	0.54 (−4.29,5.37)	0.28 (−3.83,4.39)	-		
TCM-EPS	−0.35 (−3.51,2.82)	−1.47 (−6.09,3.15)	−1.03 (−5.64,3.57)	−1.71 (−8.30,4.88)	−5.59 (−13.40,2.21)	−0.72 (−5.34,3.91)	−1.39 (−6.02,3.25)	−0.76 (−5.38,3.87)	−0.81 (−4.40,2.77)	−1.20 (−6.36,3.95)	0.21 (−2.89,3.30)	−0.05 (−2.59,2.49)	−0.33 (−5.16,4.50)	-	
CT	1.77 (−0.60,4.15)	0.65 (−3.47,4.77)	1.09 (−3.02,5.19)	0.41 (−5.84,6.66)	−3.47 (−10.99,4.04)	1.40 (−2.72,5.53)	0.73 (−3.40,4.87)	1.36 (−2.76,5.49)	1.31 (−1.61,4.22)	0.92 (−3.79,5.62)	2.32 (0.04,4.61)^%^	2.07 (0.62,3.52)^%^	1.79 (−2.57,6.14)	2.12 (0.03,4.21)^%^	-

**Figure 5:**
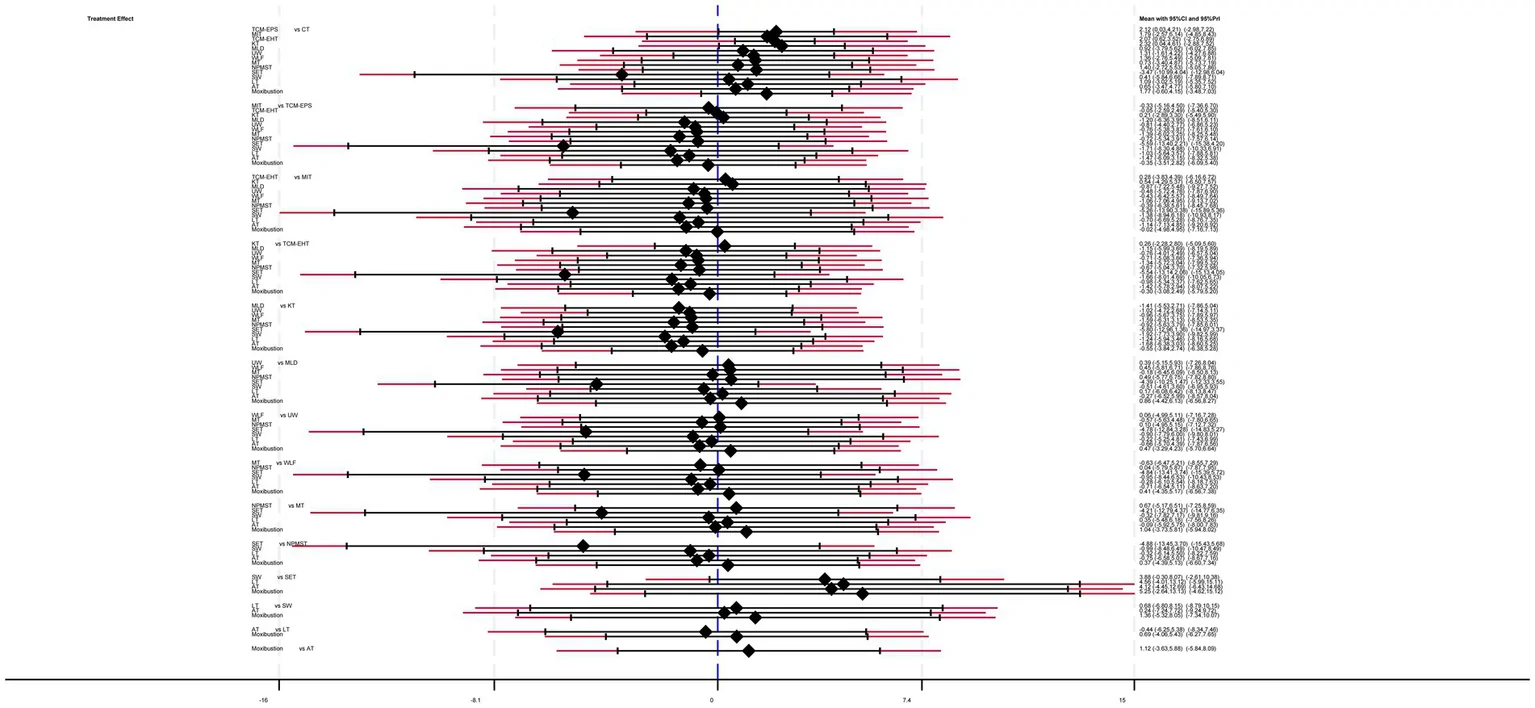
Forest plot of FMA-UE.

#### Publication bias

The comparison-adjusted funnel plot for the FMA-UE network showed some visual asymmetry, with several relatively dispersed estimates, particularly among less precise studies. Given the sparse evidence for some treatment comparisons, small-study effects or publication bias could not be excluded (Figure 4C).

### BI

#### Network geometry

A total of 18 studies reported BI outcomes involving 12 treatment strategies. Three closed loops were identified in the evidence network. The network geometry of the BI is shown in Figure 6A.

**Figure 6:**
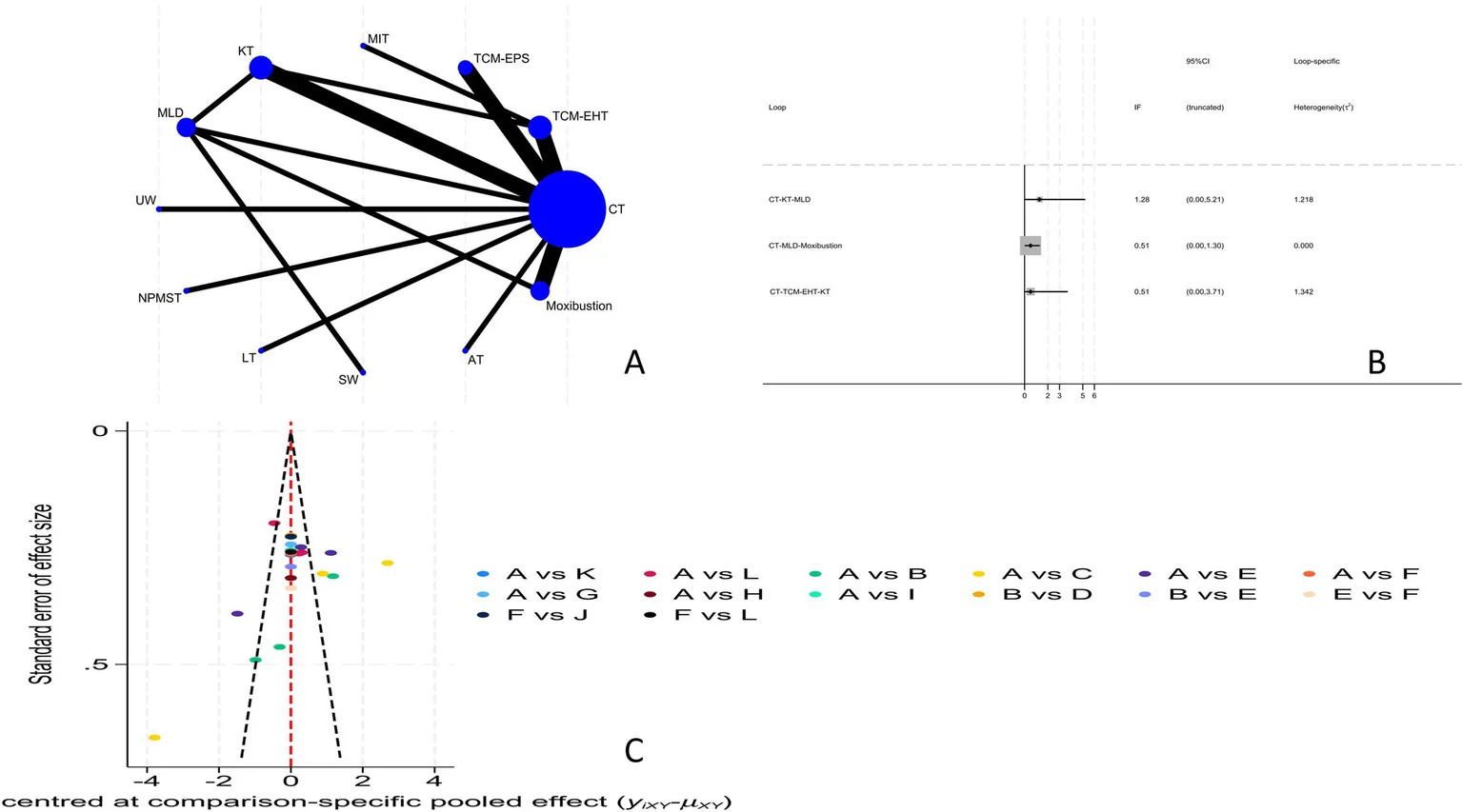
Network diagram of BI **(A)**; BI inconsistency testing **(B)**; and publication bias graph of BI **(C)**.

#### Inconsistency assessment

For BI, the 12 treatment strategies formed three triangular loops. The global inconsistency test showed no statistically significant evidence of inconsistency (*p* = 0.9294). Loop-specific analysis likewise showed no statistically significant inconsistency in the CT–KT–MLD loop (IF = 1.28, 95% CI 0.00 to 5.21), the CT–MLD–Moxibustion loop (IF = 0.51, 95% CI 0.00 to 1.30), or the CT–TCM-EHT–KT loop (IF = 0.51, 95% CI 0.00 to 3.71). Node-splitting analysis further showed no statistically significant disagreement between direct and indirect estimates for any evaluable comparison (all *p* > 0.05). Overall, no statistically significant evidence of either global or local inconsistency was detected in the BI network. The detailed results are shown in Figure 6B.

#### Network meta-analysis

A total of 18 RCTs contributed to the BI network meta-analysis, comprising 12 treatment nodes: CT, TCM-EHT, TCM-EPS, MIT, KT, MLD, UW, NPMST, LT, SW, AT, and moxibustion. Higher BI scores indicated better activities of daily living. Compared with CT, statistically significant improvements were observed with TCM-EHT (SMD = 3.64, 95% CI 1.76 to 5.51), TCM-EPS (SMD = 2.55, 95% CI 0.60 to 4.50), and KT (SMD = 1.98, 95% CI 0.27 to 3.70). In contrast, MIT, MLD, UW, NPMST, LT, SW, AT, and moxibustion did not show statistically significant differences compared with CT. Pairwise comparisons among all interventions are presented in Table 5. The network estimates with corresponding 95% confidence intervals and 95% prediction intervals are shown in Figure 7. Although TCM-EHT, TCM-EPS, and KT showed statistically significant improvements compared with CT based on the 95% confidence intervals, their corresponding 95% prediction intervals all crossed the null value (TCM-EHT: −2.46 to 9.74; TCM-EPS: −3.62 to 8.71; KT: −3.99 to 7.96), indicating substantial uncertainty regarding the effects that might be observed in future comparable studies (Figure 7).

**Table 5:** Network meta-analysis of BI.

Treatment	Moxibustion	AT	SW	LT	NPMST	UW	MLD	KT	MIT	TCM-EPS	TCM-EHT	CT
Moxibustion	-											
AT	−0.04 (−3.83,3.75)	-										
SW	0.30 (−3.89,4.50)	0.35 (−4.88,5.57)	-									
LT	−1.62 (−5.41,2.16)	−1.58 (−6.25,3.08)	−1.93 (−7.15,3.29)	-								
NPMST	−1.31 (−5.11,2.50)	−1.27 (−5.94,3.41)	−1.61 (−6.85,3.62)	0.32 (−4.36,4.99)	-							
UW	−0.56 (−4.35,3.22)	−0.52 (−5.18,4.14)	−0.87 (−6.09,4.35)	1.06 (−3.60,5.72)	0.74 (−3.93,5.42)	-						
MLD	0.31 (−2.31,2.92)	0.35 (−3.71,4.41)	0.00 (−3.29,3.29)	1.93 (−2.12,5.98)	1.61 (−2.46,5.69)	0.87 (−3.18,4.92)	-					
KT	−1.38 (−3.80,1.05)	−1.34 (−5.05,2.38)	−1.68 (−5.77,2.40)	0.25 (−3.47,3.96)	−0.07 (−3.80,3.66)	−0.81 (−4.53,2.90)	−1.68 (−4.11,0.74)	-				
MIT	−2.92 (−7.12,1.28)	−2.88 (−7.90,2.14)	−3.22 (−8.71,2.27)	−1.30 (−6.31,3.72)	−1.61 (−6.64,3.42)	−2.36 (−7.37,2.66)	−3.23 (−7.62,1.17)	−1.54 (−5.55,2.46)	-			
TCM-EPS	−1.94 (−4.64,0.75)	−1.90 (−5.73,1.93)	−2.25 (−6.74,2.25)	−0.32 (−4.15,3.51)	−0.63 (−4.48,3.21)	−1.38 (−5.21,2.45)	−2.25 (−5.31,0.81)	−0.56 (−3.16,2.03)	0.98 (−3.28,5.23)	-		
TCM-EHT	−3.03 (−5.65,-0.41)	−2.99 (−6.78,0.80)	−3.34 (−7.73,1.06)	−1.41 (−5.20,2.38)	−1.72 (−5.53,2.09)	−2.47 (−6.26,1.32)	−3.34 (−6.26,-0.42)	−1.65 (−3.94,0.63)	−0.11 (−3.40,3.18)	−1.09 (−3.79,1.61)	-	-
CT	0.61 (−1.26,2.47)	0.65 (−2.65,3.95)	0.30 (−3.75,4.35)	2.23 (−1.07,5.53)	1.92 (−1.40,5.23)	1.17 (−2.12,4.46)	0.30 (−2.06,2.66)	1.98 (0.27,3.70)^%^	3.53 (−0.26,7.31)	2.55 (0.60,4.50)^%^	3.64 (1.76,5.51)^%^	-

**Figure 7:**
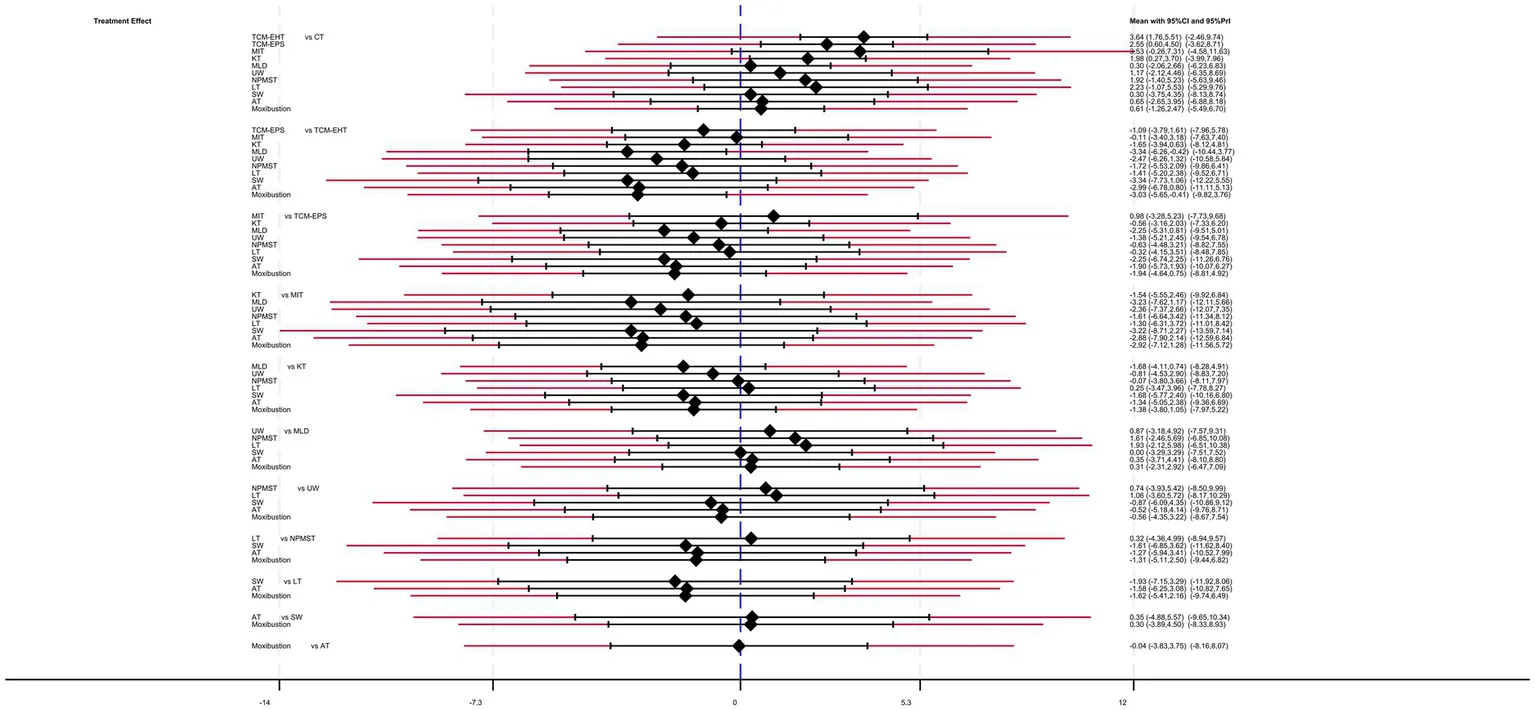
Forest plot of BI.

#### Publication bias

The comparison-adjusted funnel plot for the BI network showed some visual asymmetry, with a few relatively dispersed estimates, particularly among less precise studies. Given the limited number of studies contributing to several treatment comparisons, small-study effects or publication bias could not be excluded (Figure 6C).

### SUCRA rankings

The SUCRA-based treatment rankings varied across outcomes. For VAS, AT showed the highest SUCRA value (81.0%), followed by KT (79.4%) and moxibustion (62.9%), whereas CT had the lowest SUCRA value (10.5%). For FMA-UE, KT ranked highest (71.3%), followed by TCM-EPS (67.5%) and TCM-EHT (67.1%). These three interventions also showed statistically significant network estimates compared with CT; however, their corresponding 95% prediction intervals all crossed the null value, indicating uncertainty regarding the effects that might be observed in future comparable studies. For BI, TCM-EHT showed the highest SUCRA value (86.8%), followed by MIT (79.2%) and TCM-EPS (69.4%). Notably, although MIT ranked second for BI, its network estimate versus CT was not statistically significant. Overall, treatment rankings differed across outcomes, suggesting that the relative performance of interventions depended on the clinical outcome evaluated. High SUCRA values should therefore not be interpreted as definitive evidence of clinical superiority. Treatment rankings were interpreted alongside the corresponding effect estimates, 95% confidence intervals, 95% prediction intervals, and inconsistency assessments. The SUCRA plots are presented in Figure 8, and detailed ranking probabilities are provided in Supplementary Table 5.

**Figure 8:**
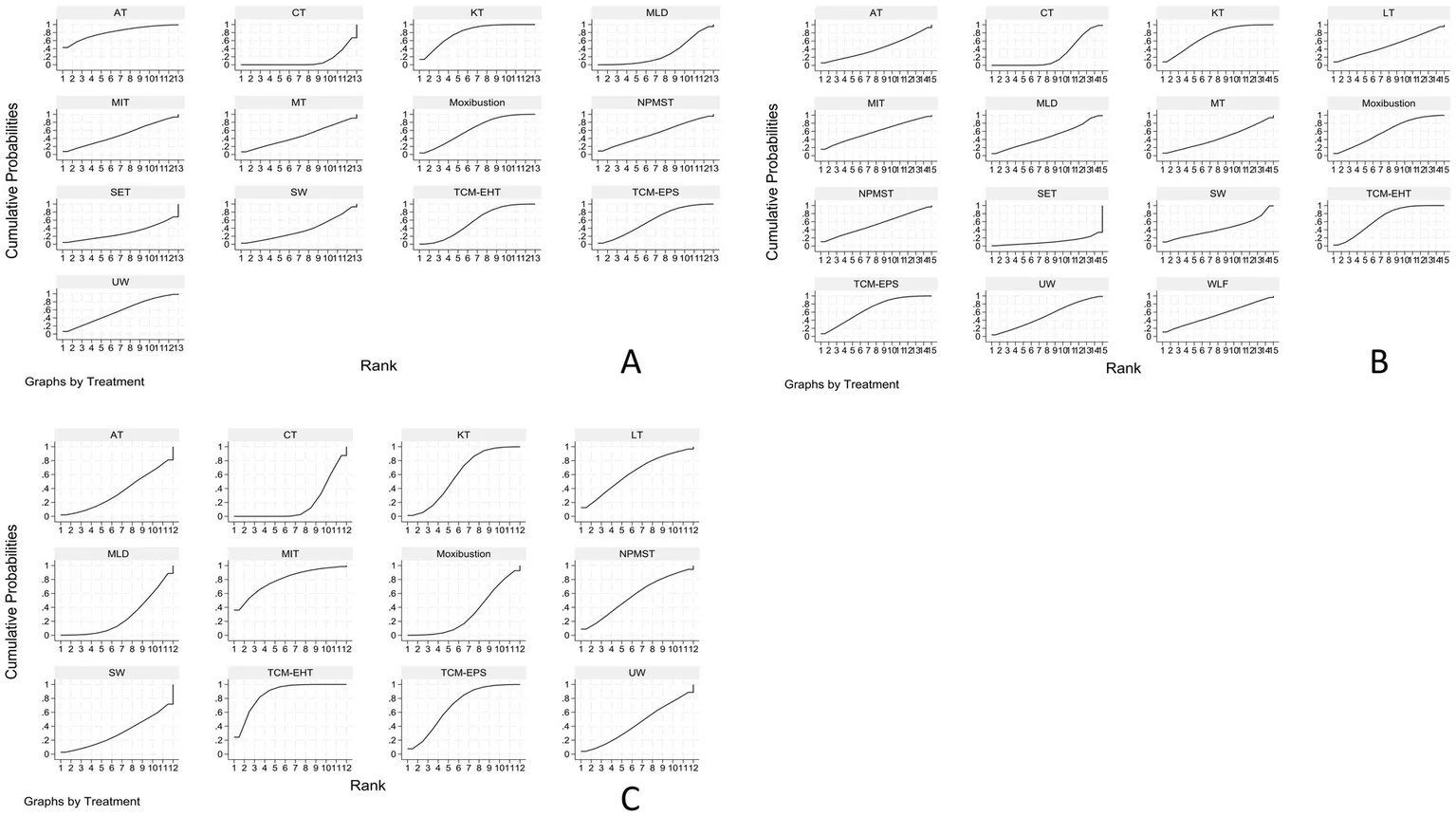
SUCRA **(A)** VAS; **(B)** FMA-UE; **(C)** BI.

### Certainty of evidence assessment

The certainty of evidence from the network meta-analysis was assessed using the CINeMA framework across six domains: within-study bias, reporting bias, indirectness, imprecision, heterogeneity, and incoherence. The certainty of evidence was assessed for prespecified clinically relevant comparisons against CT that directly informed the main conclusions of this review. Overall, the certainty of evidence ranged from moderate to low. For the VAS outcome, moderate-certainty evidence suggested potential benefits of AT, KT, and moxibustion compared with CT, whereas the certainty of evidence for TCM-EPS and TCM-EHT was rated as low due to concerns regarding indirectness, imprecision, and, for TCM-EHT, incoherence between direct and indirect evidence. For FMA-UE, the certainty of evidence for KT, TCM-EPS, and TCM-EHT compared with CT was rated as low, mainly because of concerns regarding risk of bias, imprecision, and heterogeneity. For BI, the certainty of evidence for TCM-EHT, TCM-EPS, KT, and MIT compared with CT was also rated as low, primarily due to concerns regarding risk of bias, heterogeneity, and imprecision for some comparisons. Although several interventions demonstrated statistically significant improvements in specific outcomes, the certainty assessment indicated that confidence in these estimates remains limited. Therefore, the comparative effectiveness of non-invasive interventions for stage I SHS should be interpreted cautiously, and treatment decisions should not rely solely on statistical significance or SUCRA rankings. Detailed CINeMA assessments for the prespecified clinically relevant comparisons are presented in Supplementary Table 6.

### Adverse events

Adverse events were poorly reported across the included studies. Among the 36 included RCTs, only two studies provided explicit information on adverse events. Chen Rongqun et al. (13) reported two cases of rash in the TCM-EHT group. Qiao Xiuqi et al. (28) reported one case of edema, two cases of infection, and four cases of gastrointestinal abnormalities in the CT group, and one case of gastrointestinal discomfort in the TCM-EHT group. The remaining included studies did not report adverse events. Owing to the limited and inconsistent reporting of safety outcomes, no quantitative synthesis or comparative assessment of adverse events was performed.

## Discussion

In this study, 14 active non-invasive interventions were evaluated across the included networks, with CT serving as the reference treatment. The composition of the networks differed according to the outcome assessed. The findings varied across the three clinical outcomes of pain intensity (VAS), upper limb motor function (FMA-UE), and activities of daily living (BI), and no intervention showed consistent evidence of superiority across all outcomes. For pain intensity, AT, KT, moxibustion, TCM-EPS, and TCM-EHT were associated with statistically significant reductions in VAS scores compared with CT. For upper limb motor function, KT, TCM-EPS, and TCM-EHT showed statistically significant improvements in FMA-UE scores, whereas TCM-EHT, TCM-EPS, and KT showed statistically significant improvements in BI. However, the 95% prediction intervals for all of these statistically significant comparisons crossed the null value, indicating substantial uncertainty regarding the range of effects that might be observed in future comparable studies. Collectively, these findings suggest that the relative effects of non-invasive interventions differed according to the clinical outcome assessed. Nevertheless, the results should be interpreted cautiously, because statistical significance and treatment-ranking probabilities do not constitute definitive evidence of clinical superiority, particularly in the presence of imprecision, sparse evidence for several comparisons, localized incoherence in the VAS network, and methodological limitations of the included trials.

### Clinical outcomes

In this study, VAS, FMA-UE, and BI were selected as key clinical outcomes reflecting pain intensity, upper limb motor function, and activities of daily living, respectively. Patients with stage I SHS commonly present with concurrent pain, impaired upper limb motor function, and limitations in activities of daily living; therefore, reliance on a single outcome measure may not adequately capture the multidimensional effects of rehabilitation interventions. VAS is widely used as a patient-reported measure of pain intensity (48), whereas FMA-UE provides a standardized assessment of upper limb motor impairment and motor recovery after stroke (49). BI, in contrast, reflects independence in activities of daily living and functional performance in everyday activities (50). By jointly considering these complementary outcomes, the present study evaluated the comparative effects of non-invasive interventions for stage I SHS across pain relief, upper limb motor recovery, and activities of daily living. Nevertheless, statistically significant differences on these scales should not automatically be interpreted as clinically meaningful benefits. Where established minimally important difference thresholds are available and applicable, the magnitude of treatment effects should also be interpreted in relation to those thresholds.

### Stage-specific considerations for stage I SHS

Stage I SHS is characterized predominantly by pain, hand oedema, vasomotor or autonomic disturbances, sensory abnormalities, and progressive limitation of upper-limb movement. Its early pathophysiological features are thought to involve local inflammatory and neurogenic responses, peripheral and central pain sensitization, vasomotor and autonomic dysfunction, and biomechanical abnormalities associated with post-stroke motor impairment (51). These processes may interact and contribute to a vicious cycle of pain, swelling, reduced movement, and further functional decline. Accordingly, treatment during this stage generally emphasizes early protection of the affected limb, reduction of oedema and pain, maintenance of joint mobility, and facilitation of safe upper-limb activity (52, 53). This stage-specific therapeutic focus provides a clinical rationale for evaluating non-invasive interventions separately in patients with stage I SHS rather than pooling different disease stages with potentially distinct manifestations and treatment priorities.

### Pain-related outcomes

The present study demonstrated differences in the estimated effects of non-invasive interventions on pain intensity. Compared with CT, AT, KT, moxibustion, TCM-EPS, and TCM-EHT were associated with statistically significant reductions in VAS scores. Previous reviews have proposed that non-invasive external interventions may alleviate pain through mechanisms involving sensory stimulation, local circulatory changes, and modulation of soft-tissue or oedema-related processes (54, 55). Herbal external therapies have also been used clinically in combination with rehabilitation for post-stroke SHS (56); however, the present NMA cannot determine whether the observed effects of TCM-EHT were mediated by transdermal absorption, anti-inflammatory pathways, microcirculatory changes, or other mechanisms. Kinesiology taping has likewise been investigated in post-stroke rehabilitation, although previous systematic evidence has indicated considerable uncertainty regarding its effects on pain and functional outcomes (57). Therefore, potential biological mechanisms underlying the observed treatment effects should be regarded as hypotheses rather than mechanisms directly established by the present analysis. MIT, MLD, MT, UW, NPMST, SET, and SW did not show statistically significant differences from CT. However, the absence of statistical significance should not be interpreted as evidence of no analgesic effect, particularly because several estimates were imprecise. Moreover, although AT, KT, moxibustion, TCM-EPS, and TCM-EHT showed statistically significant mean effects, their corresponding 95% prediction intervals all crossed the null value, indicating substantial uncertainty regarding the effects that might be observed in future comparable studies. Accordingly, these pain-related findings should be interpreted cautiously rather than as definitive evidence of comparative analgesic superiority. Additional caution is warranted because VAS is a subjective patient-reported outcome that may be particularly susceptible to expectation and performance effects. Participant blinding was generally infeasible or inadequately reported for the physical and rehabilitative interventions included in this review, which may have influenced self-reported pain ratings. Consequently, the magnitude of the observed analgesic effects may have been overestimated. Taken together with the wide prediction intervals and imprecision in several comparisons, the VAS findings should be regarded as suggestive rather than definitive evidence of comparative analgesic efficacy.

### Functional outcomes

Regarding functional outcomes, the present findings showed broadly similar patterns for upper limb motor function and activities of daily living. Compared with CT, TCM-EPS, TCM-EHT, and KT were associated with statistically significant improvements in FMA-UE scores. The same three interventions also showed statistically significant improvements in BI, suggesting that their potential benefits may extend from motor impairment to performance in activities of daily living. Nevertheless, the magnitude of these standardized effects should be interpreted cautiously, particularly because several estimates were large and accompanied by relatively wide confidence intervals. Previous evidence has suggested potential benefits of TCM-based interventions for post-stroke motor rehabilitation, although the certainty and methodological quality of the available evidence remain limited (58). The present findings for TCM-EPS and TCM-EHT are broadly compatible with this literature; however, the mechanisms underlying these effects were not directly evaluated in the included trials or in the present NMA and should therefore not be regarded as established explanations. KT also showed statistically significant improvements in both FMA-UE and BI, suggesting a potentially favorable functional effect, although the precision and robustness of these estimates remain limited. Importantly, although TCM-EPS, TCM-EHT, and KT reached statistical significance for both functional outcomes based on their 95% confidence intervals, the corresponding 95% prediction intervals all crossed the null value. This indicates substantial uncertainty regarding whether similar effects would be reproduced in future comparable studies. In addition, because SMDs were used to accommodate differences in FMA and BI scoring and reporting across studies, the magnitude of the pooled effects should not be interpreted directly as changes in the original scale units or as established clinically important differences. Therefore, the functional findings should be regarded as suggestive evidence of potential benefit rather than definitive evidence of functional superiority.

### Inconsistency and robustness of the network estimates

No statistically significant evidence of global or loop-specific inconsistency was detected for the FMA-UE or BI networks, and node-splitting analyses likewise showed no statistically significant disagreement between direct and indirect evidence for any evaluable comparison. These findings provide some support for the coherence of the functional-outcome networks, although the absence of statistically significant inconsistency should not be interpreted as proof of complete consistency, particularly because several comparisons were sparsely informed and some indirect estimates were imprecise. For the VAS network, no statistically significant evidence of global or loop-specific inconsistency was detected; however, node-splitting analysis identified localized disagreement between direct and indirect evidence for the CT versus TCM-EHT comparison. Leave-one-study-out sensitivity analyses did not identify a single trial that clearly accounted for this discrepancy. Exclusion of several individual studies attenuated the inconsistency signal to borderline non-significance, whereas exclusion of others did not materially reduce it. In one analysis, removal of a multi-arm study substantially altered the network geometry and rendered the indirect estimate highly imprecise, limiting interpretation of the corresponding node-splitting result. These findings suggest that the localized incoherence was unlikely to be attributable to a single influential trial and may instead reflect broader clinical or methodological heterogeneity across the evidence pathways. Potential contributors include variation in intervention protocols, treatment intensity and duration, baseline severity, concomitant rehabilitation, and other poorly reported effect modifiers. Because these characteristics were not consistently reported across studies, formal exploration through subgroup analysis or meta-regression was not feasible. Accordingly, the VAS comparison between CT and TCM-EHT should be interpreted with additional caution, whereas the absence of statistically significant inconsistency in the FMA-UE and BI networks does not eliminate uncertainty arising from sparse evidence and imprecision.

### SUCRA

In this study, SUCRA rankings provided complementary information to the effect estimates derived from the network meta-analysis and were interpreted together with the corresponding effect sizes, 95% CIs, 95% prediction intervals, inconsistency assessments, and certainty of evidence rather than as standalone evidence of clinical superiority. For VAS, AT showed the highest SUCRA value (81.0%), followed by KT (79.4%) and moxibustion (62.9%). AT and KT were both associated with statistically significant reductions in VAS scores compared with CT; however, their 95% prediction intervals crossed the null value, indicating uncertainty regarding the effects that might be observed in future comparable studies. Thus, even when a high ranking coincides with a statistically significant mean effect, the ranking itself should not be interpreted as evidence of stable or clinically meaningful superiority. For FMA-UE, KT showed the highest SUCRA value (71.3%), followed by TCM-EPS (67.5%) and TCM-EHT (67.1%). All three interventions were associated with statistically significant improvements compared with CT, but their corresponding 95% prediction intervals crossed the null value. This indicates that favorable ranking probabilities and statistically significant pooled effects were accompanied by substantial uncertainty regarding the reproducibility of these effects in future studies. For BI, TCM-EHT ranked highest (86.8%), followed by MIT (79.2%) and TCM-EPS (69.4%). Notably, although MIT ranked second, its network estimate versus CT was not statistically significant. This illustrates that treatment-ranking probabilities and evidence of treatment effect are distinct statistical concepts and should not be interpreted interchangeably. Overall, SUCRA rankings may help describe the relative positioning of interventions within each outcome network, but they should not be used alone to guide clinical treatment selection. Rankings should instead be considered alongside effect estimates, 95% CIs, 95% prediction intervals, network incoherence, certainty of evidence, and clinical relevance.

### Limitations

To the best of our knowledge, this study is among the first network meta-analyses to compare multiple non-invasive interventions specifically for stage I SHS. Nevertheless, several limitations should be acknowledged. First, clinical and methodological heterogeneity existed across the included studies, including differences in sample size, intervention protocols, treatment frequency and duration, baseline disease severity, outcome assessment time points, and components of conventional treatment. Although treatment nodes were defined according to their predominant therapeutic mechanisms and modes of delivery, some residual clinical heterogeneity may remain within broader categories such as TCM-EPS and TCM-EHT. Consequently, the transitivity assumption may not have been fully satisfied. Second, direct comparative evidence was sparse for several interventions, and some network estimates relied substantially on indirect evidence. This was reflected by wide confidence intervals and prediction intervals for several comparisons, as well as very large standard errors for some indirect estimates. The resulting imprecision limits confidence in both comparative effect estimates and treatment rankings. Third, although no statistically significant evidence of global or loop-specific inconsistency was detected in the VAS network, node-splitting analysis identified localized disagreement between direct and indirect evidence for the CT versus TCM-EHT comparison. Leave-one-study-out sensitivity analyses did not identify a single study that clearly accounted for this discrepancy, suggesting that broader clinical or methodological heterogeneity across evidence pathways may have contributed. In contrast, no statistically significant evidence of global or local inconsistency was detected in the FMA-UE or BI networks. Nevertheless, the absence of statistically significant inconsistency should not be interpreted as proof of complete coherence, particularly given the sparse evidence and limited precision of some comparisons. Fourth, some continuous outcomes required calculation of change-score standard deviations using an assumed correlation coefficient. Although the extracted data and analytical datasets were carefully rechecked, uncertainty related to data transformation and assumptions used in deriving change scores could not be completely eliminated. Fifth, many included RCTs were small, single-center studies with limited or unclear reporting of allocation concealment, blinding, and prospective trial registration. These methodological limitations may increase the risk of bias and may also contribute to exaggerated treatment effects or small-study effects, particularly for subjective outcomes such as VAS. Sixth, all included studies were conducted in China, which may limit the generalisability of the findings to populations, rehabilitation practices, and healthcare systems in other settings. Seventh, adverse events and other clinically relevant considerations, including treatment burden, acceptability, resource requirements, therapist expertise, and feasibility, were insufficiently reported. Consequently, the comparative safety and practical applicability of the interventions could not be adequately assessed. Finally, several statistically significant comparisons were accompanied by 95% prediction intervals that crossed the null value, and treatment rankings were not always concordant with statistically significant treatment effects. Therefore, SUCRA rankings should be interpreted together with effect estimates, 95% confidence intervals, prediction intervals, network incoherence, certainty of evidence, and clinical relevance rather than being used alone to guide treatment selection. Future multicentre, adequately powered, prospectively registered RCTs with standardized intervention protocols, consistent outcome definitions and assessment time points, rigorous allocation concealment and blinding where feasible, and systematic reporting of safety and implementation-related outcomes are warranted.

## Conclusion

This network meta-analysis compared multiple non-invasive interventions for stage I SHS across pain intensity, upper limb motor function, and activities of daily living. The findings suggest that the relative effects of different interventions varied according to the outcome assessed. Compared with CT, AT, KT, moxibustion, TCM-EPS, and TCM-EHT were associated with statistically significant reductions in VAS scores; TCM-EPS, TCM-EHT, and KT were associated with statistically significant improvements in FMA-UE; and TCM-EHT, TCM-EPS, and KT were associated with statistically significant improvements in BI. However, the corresponding 95% prediction intervals for all of these statistically significant comparisons crossed the null value, indicating uncertainty regarding the reproducibility of these effects in future comparable studies. No statistically significant evidence of global inconsistency was detected across the three outcome networks. However, node-splitting analysis identified localized disagreement between direct and indirect evidence for the CT versus TCM-EHT comparison in the VAS network. Therefore, the findings should be interpreted cautiously and should not be regarded as definitive evidence of clinical superiority. Treatment selection for stage I SHS should consider the predominant clinical problems and rehabilitation goals of individual patients, together with the magnitude and certainty of treatment effects, safety, feasibility, and resource requirements, rather than relying on SUCRA rankings alone. Further adequately powered, multicentre, prospectively registered RCTs with rigorous methodology and systematic reporting of safety and implementation-related outcomes are needed to clarify the comparative benefits and harms of non-invasive interventions for stage I SHS.

## Data Availability

The original contributions presented in the study are included in the article/Supplementary material, further inquiries can be directed to the corresponding author.

## Glossary

AT: Air pressure therapy
BI: Barthel Index
CRPS: Complex regional pain syndrome
CT: Conventional therapy
TCM-EHT: Traditional Chinese Medicine External Herbal Therapy
TCM-EPS: Traditional Chinese Medicine External Physical Stimulation
FMA-UE: Fugl–Meyer Assessment–Upper Extremity
KT: Kinesiology taping
LT: Laser therapy
MLD: Manual lymphatic drainage
MIT: Motor imagery therapy
MT: Mirror therapy
NPMST: Negative pressure mechanical stimulation therapy
RCT: Randomized controlled trial
SET: Suspension exercise therapy
SHS: Shoulder–hand syndrome
SUCRA: Surface under the cumulative ranking curve
SW: Shock wave therapy
UW: Ultrashort wave
VAS: Visual Analog Scale
WLF: Warm low-frequency therapy
