# Supplementary material for: Comparative effectiveness of non-invasive interventions for stage I post-stroke shoulder–hand syndrome: a network meta-analysis of randomized controlled trials
Source: Front Neurol. 2026 Sep 23;17:1932931. doi: 10.3389/fneur.2026.1932931 (PMC13642199; doi:10.3389/fneur.2026.1932931)
Supplement: Supplementary file 1 [file Supplementary_file_1.DOC]

**Pubmed**

#1 (Shoulder hand syndrome[MeSH Terms]) OR ((Reflex Sympathetic Dystrophy[Title/Abstract]) OR (Dystrophies, Reflex Sympathetic[Title/Abstract]) OR (Reflex Sympathetic Dystrophies[Title/Abstract]) OR (Sympathetic Dystrophies, Reflex[Title/Abstract]) OR (Sympathetic Dystrophy, Reflex[Title/Abstract]) OR (CRPS Type I[Title/Abstract]) OR (CRPS Type Is[Title/Abstract] OR Type I, CRPS[Title/Abstract]) OR (Complex Regional Pain Syndrome, Type I[Title/Abstract]) OR (Pain Syndrome Type I, Regional, Complex[Title/Abstract]) OR (Sudek Atrophy[Title/Abstract] OR Atrophy, Sudek[Title/Abstract]) OR (Sympathetic Reflex Dystrophia[Title/Abstract]) OR (Reflex Dystrophia, Sympathetic[Title/Abstract]) OR (Sympathetic Reflex Dystrophias[Title/Abstract]) OR (Syndrome, Reflex Sympathetic Dystrophy[Title/Abstract]) OR (Type I Complex Regional Pain Syndrome[Title/Abstract]) OR (Sudek's Atrophy[Title/Abstract]) OR (Atrophies, Sudek's[Title/Abstract]) OR (Atrophy, Sudek's[Title/Abstract]) OR (Sudek's Atrophies[Title/Abstract]) OR (Sudeks Atrophy[Title/Abstract]) OR (Pain Syndrome Type I, Complex Regional[Title/Abstract]) OR (Algodystrophic Syndrome[Title/Abstract]) OR (Syndrome, Algodystrophic[Title/Abstract]) OR (Algodystrophy[Title/Abstract]) OR (Algodystrophies[Title/Abstract]) OR (Cervical Sympathetic Dystrophy[Title/Abstract]) OR (Cervical Sympathetic Dystrophies[Title/Abstract]) OR (Dystrophies, Cervical Sympathetic[Title/Abstract]) OR (Dystrophy, Cervical Sympathetic[Title/Abstract]) OR (Sympathetic Dystrophies, Cervical[Title/Abstract]) OR (Sympathetic Dystrophy, Cervical[Title/Abstract]) OR (Shoulder-Hand Syndrome[Title/Abstract]) OR (Shoulder Hand Syndrome[Title/Abstract]) OR (Shoulder-Hand Syndromes[Title/Abstract]) OR (Syndrome, Shoulder-Hand[Title/Abstract]) OR (Syndromes, Shoulder-Hand[Title/Abstract]) OR (Shoulder hand syndrome[Title/Abstract]))

#2 (clinical[tiab] AND trial[tiab]) OR "clinical trials as topic"[mesh] OR "clinical trial"[pt] OR random*[tiab] OR "random allocation"[mesh] OR "therapeutic use"[sh]

#3(Stroke[Title/Abstract] OR Strokes[Title/Abstract] OR Cerebrovascular Accident[Title/Abstract] OR Cerebrovascular Accidents[Title/Abstract] OR Cerebrovascular Apoplexy[Title/Abstract] OR Apoplexy, Cerebrovascular[Title/Abstract] OR Vascular Accident, Brain[Title/Abstract] OR Cerebrovascular Stroke[Title/Abstract] OR Cerebrovascular Strokes[Title/Abstract] OR Stroke, Cerebrovascular[Title/Abstract]) OR (stroke[MeSH Terms])

#4

#1 AND #2 AND #3

87

Embase

#1('strokes':ab,ti OR 'stroke':ab,ti OR 'cerebrovascular accidents':ab,ti OR 'cerebrovascular apoplexy':ab,ti OR 'apoplexy, cerebrovascular':ab,ti OR 'vascular accident, brain':ab,ti OR 'cerebrovascular stroke':ab,ti OR 'cerebrovascular strokes':ab,ti OR 'stroke, cerebrovascular':ab,ti OR 'cerebrovascular accident':ab,ti)

#2('Reflex Sympathetic Dystrophy':ab,ti OR 'Dystrophies, Reflex Sympathetic':ab,ti OR 'Reflex Sympathetic Dystrophies':ab,ti OR 'Sympathetic Dystrophies, Reflex':ab,ti OR 'Sympathetic Dystrophy, Reflex':ab,ti OR 'CRPS Type I':ab,ti OR 'CRPS Type Is':ab,ti OR 'Type I, CRPS':ab,ti OR 'Complex Regional Pain Syndrome, Type I':ab,ti OR 'Pain Syndrome Type I, Regional, Complex':ab,ti OR 'Sudek Atrophy':ab,ti OR 'Sympathetic Reflex Dystrophia':ab,ti OR 'Reflex Dystrophia, Sympathetic':ab,ti OR 'Sympathetic Reflex Dystrophias':ab,ti OR 'Syndrome, Reflex Sympathetic Dystrophy':ab,ti OR 'Type I Complex Regional Pain Syndrome':ab,ti OR 'Sudeks Atrophy':ab,ti OR 'Pain Syndrome Type I, Complex Regional':ab,ti OR 'Algodystrophic Syndrome':ab,ti OR 'Syndrome, Algodystrophic':ab,ti OR 'Algodystrophy':ab,ti OR 'Algodystrophies':ab,ti OR 'Cervical Sympathetic Dystrophy':ab,ti OR 'Cervical Sympathetic Dystrophies':ab,ti OR 'Dystrophies, Cervical Sympathetic':ab,ti OR 'Dystrophy, Cervical Sympathetic':ab,ti OR 'Sympathetic Dystrophies, Cervical':ab,ti OR 'Sympathetic Dystrophy, Cervical':ab,ti OR 'Shoulder-Hand Syndrome':ab,ti OR 'Shoulder Hand Syndrome':ab,ti OR 'Shoulder-Hand Syndromes':ab,ti OR 'Syndrome, Shoulder-Hand':ab,ti OR 'Syndromes, Shoulder-Hand':ab,ti OR 'Shoulder hand syndrome':ab,ti)

#3('randomized controlled trial'/exp OR 'controlled clinical trial'/exp OR randomized:ti,ab OR placebo:ti,ab OR 'drug therapy':lnk OR randomly:ti,ab OR trial:ti,ab OR groups:ti,ab)

#4

#1 AND #2 AND #3

135

考克兰

#1(Strokes):ti,ab,kw OR (stroke):ti,ab,kw OR (Cerebrovascular Accident):ti,ab,kw OR (Cerebrovascular Accidents):ti,ab,kw OR (Cerebrovascular Apoplexy):ti,ab,kw OR (Apoplexy, Cerebrovascular):ti,ab,kw OR (Vascular Accident, Brain):ti,ab,kw OR (Cerebrovascular Stroke):ti,ab,kw OR (Cerebrovascular Strokes):ti,ab,kw OR (Stroke, Cerebrovascular):ti,ab,kw

#2 (Reflex Sympathetic Dystrophy):ti,ab,kw OR (Dystrophies, Reflex Sympathetic):ti,ab,kw OR (Reflex Sympathetic Dystrophies):ti,ab,kw OR (Sympathetic Dystrophies, Reflex):ti,ab,kw OR (Sympathetic Dystrophy, Reflex):ti,ab,kw OR (CRPS Type I):ti,ab,kw OR (CRPS Type Is):ti,ab,kw OR (Type I, CRPS):ti,ab,kw OR (Complex Regional Pain Syndrome, Type I):ti,ab,kw OR (Pain Syndrome Type I, Regional, Complex):ti,ab,kw OR (Sudek Atrophy):ti,ab,kw OR (Atrophy, Sudek):ti,ab,kw OR (Sympathetic Reflex Dystrophia):ti,ab,kw OR (Reflex Dystrophia, Sympathetic):ti,ab,kw OR (Sympathetic Reflex Dystrophias):ti,ab,kw OR (Syndrome, Reflex Sympathetic Dystrophy):ti,ab,kw OR (Type I Complex Regional Pain Syndrome):ti,ab,kw OR (Sudek's Atrophy):ti,ab,kw OR (Atrophies, Sudek's):ti,ab,kw OR (Atrophy, Sudek's):ti,ab,kw OR (Sudek's Atrophies):ti,ab,kw OR (Sudeks Atrophy):ti,ab,kw OR (Pain Syndrome Type I, Complex Regional):ti,ab,kw OR (Algodystrophic Syndrome):ti,ab,kw OR (Syndrome, Algodystrophic):ti,ab,kw OR (Algodystrophy):ti,ab,kw OR (Algodystrophies):ti,ab,kw OR (Cervical Sympathetic Dystrophy):ti,ab,kw OR (Cervical Sympathetic Dystrophies):ti,ab,kw OR (Dystrophies, Cervical Sympathetic):ti,ab,kw OR (Dystrophy, Cervical Sympathetic):ti,ab,kw OR (Sympathetic Dystrophies, Cervical):ti,ab,kw OR (Sympathetic Dystrophy, Cervical):ti,ab,kw OR (Shoulder-Hand Syndrome):ti,ab,kw OR (Shoulder Hand Syndrome):ti,ab,kw OR (Shoulder-Hand Syndromes):ti,ab,kw OR (Syndrome, Shoulder-Hand):ti,ab,kw OR (Syndromes, Shoulder-Hand):ti,ab,kw OR (Shoulder hand syndrome):ti,ab,kw

#3

#1 AND #2

162

**CNKI**

TKA=('卒中'+'脑缺血'+'脑梗死'+'脑出血'+'中风') AND TKA=('肩手综合征'+'反射性交感神经营养不良'+'复杂性区域疼痛综合征Ⅰ型'+'SHS'+'RSD') AND TKA=('随机')

1493

**WANFANG**

主题:(卒中 or 脑缺血 or 脑梗死 or 脑出血 or 中风) and 主题:(肩手综合征 or 反射性交感神经营养不良 or 复杂性区域疼痛综合征Ⅰ型 or SHS or RSD) and 主题:(随机)

2017

**CBM**

("脑梗死"[常用字段:智能] OR "脑出血"[常用字段:智能] OR "卒中"[常用字段:智能] OR "中风"[常用字段:智能] OR "脑缺血"[常用字段:智能]) AND ("肩手综合征"[常用字段:智能] OR "反射性交感神经营养不良"[常用字段:智能] OR "复杂性区域疼痛综合征Ⅰ型"[常用字段:智能] OR "SHS"[常用字段:智能] OR "RSD"[常用字段:智能]) AND "随机"[常用字段:智能]

1620

**WEIPU**

(U=中风 OR U=卒中 OR U=脑梗死 OR U=脑缺血 OR U=脑出血) AND (U=肩手综合征 OR U=反射性交感神经营养不良 OR U=复杂性区域疼痛综合征Ⅰ型 OR U=SHS OR U=RSD) AND U=随机

2002
